# Continuous-flow Heck synthesis of 4-methoxybiphenyl and methyl 4-methoxycinnamate in supercritical carbon dioxide expanded solvent solutions

**DOI:** 10.3762/bjoc.9.325

**Published:** 2013-12-17

**Authors:** Phei Li Lau, Ray W K Allen, Peter Styring

**Affiliations:** 1 Department of Chemical & Biological Engineering, Sir Robert Hadfield Building, The University of Sheffield, Sheffield S7 2GA, United Kingdom; 2 Department of Chemical and Environmental Engineering, The University of Nottingham, Malaysia Campus, Jalan Broga, 43500 Semenyih, Selangor Darul Shsan, Malaysia

**Keywords:** continuous flow, flow chemistry, Heck, palladium, supercritical carbon dioxide

## Abstract

The palladium metal catalysed Heck reaction of 4-iodoanisole with styrene or methyl acrylate has been studied in a continuous plug flow reactor (PFR) using supercritical carbon dioxide (scCO_2_) as the solvent, with THF and methanol as modifiers. The catalyst was 2% palladium on silica and the base was diisopropylethylamine due to its solubility in the reaction solvent. No phosphine co-catalysts were used so the work-up procedure was simplified and the green credentials of the reaction were enhanced. The reactions were studied as a function of temperature, pressure and flow rate and in the case of the reaction with styrene compared against a standard, stirred autoclave reaction. Conversion was determined and, in the case of the reaction with styrene, the isomeric product distribution was monitored by GC. In the case of the reaction with methyl acrylate the reactor was scaled from a 1.0 mm to 3.9 mm internal diameter and the conversion and turnover frequency determined. The results show that the Heck reaction can be effectively performed in scCO_2_ under continuous flow conditions with a palladium metal, phosphine-free catalyst, but care must be taken when selecting the reaction temperature in order to ensure the appropriate isomer distribution is achieved. Higher reaction temperatures were found to enhance formation of the branched terminal alkene isomer as opposed to the linear *trans*-isomer.

## Introduction

The use of cross-coupling reactions between organometallic reagents and organic halides as a straightforward method of carbon–carbon bond formation has gained much popularity over the past three decades. The development of these cross-coupling methods has undoubtedly revolutionalised the protocols for the construction of natural products, building blocks for supramolecular chemistry, self-assembly of organic materials and polymers, and lead compounds in medicinal chemistry from simpler entities [1]. These include the Suzuki, Kumada, and Heck reactions. Among these, the palladium-catalysed Heck reaction [2] is one of the most explored families of such reactions. In the Heck reactions, a vinylic hydrogen is replaced by a vinyl, aryl or benzyl group through reaction with a halide compound. This reaction has been recognised as an indispensably simple yet effective method for molecular structure elaboration. The Heck reaction is a useful component of the chemist’s toolkit as it allows carbon chain extension, with the addition of an alkene motif, using catalytic C–C bond formation chemistry. Traditionally, Heck reactions have been performed using homogeneous palladium catalysts with added phosphines, either as free reagents, coordinated discrete ligands or built into the ligand system itself. In recent years there has been a drive to develop phosphine-free catalysts and systems to ease separation and reduce both cost and product contamination [3]. Further efforts have focused on the development of heterogeneous catalysts or immobilised homogeneous catalysts, particularly for use in continuous flow reactor systems [3–6].

The yield and selectivity of Heck reactions are profoundly influenced by a number of variables, such as the nature of the base, solvent, catalyst and operating conditions. The addition of base is necessary to neutralise the halide acid formed in the reaction, which would otherwise become detrimental to the reaction system. A wide array of bases, organic and inorganic, has been investigated. Among them, tertiary amines such as triethylamine (Et_3_N), tributylamine (Bu_3_N) and alkali salts like carbonates, acetates or phosphates play crucial roles [7]. Solubility of the base and its basicity in the appropriate solvents are criteria that need to be considered.

Traditionally, most Heck reactions are carried out in polar aprotic solvents, such as *N,N*-dimethylformamide (DMF), 1-methyl-2-pyrrolidone (NMP) and *N,N*-dimethylacetamide (DMA), though other organic solvents, such as toluene, hexane, methanol and ethanol have been utilised [6]. In addition, the reactions have been carried out in biphasic mode using a mixture of solvents, such as ethylene glycol and toluene [8]. The drive to carry out reactions in environmentally benign media also led to studies being carried out in “green” (environmentally friendly) solvents, such as aqueous media, some supercritical fluids (SCFs), ionic liquids and even “solventless” systems [9–12]. Pioneering work using SCFs as reaction media was carried out independently in the late 1990s [13–14] using a palladium complex with fluorinated phosphine ligands. Most of the published results utilising supercritical fluid media have been based on homogeneously-catalysed stirred reactions, using custom-built constant volume high pressure autoclaves. Naturally this triggers the question about the possibility of using continuous reactions, an issue which this paper addresses. For continuous flow systems the catalyst stability is important as it must be used over numerous runs without catalysts degradation, metal leaching or reduction in activity through impurity deposition [12,15]. Virtually all forms of palladium have been used as catalysts for the Heck reaction. A wide array of combinations depending on the components of the catalytic system is possible, for example the catalyst can present as nanoparticles, ligand-less, unsupported or supported among other forms [16].

Practically speaking, heterogeneous catalysts provide benefits such as ease of separation and recovery from the reaction mixture. In general, heterogeneous catalysts tend to be more robust and stable under harsh reaction conditions, such as elevated temperatures and can be easily stored over longer periods, which in turn provides the possibility of by-passing the use of expensive, sensitive ligands and inert atmospheres [17]. The catalysts can be reused and recycled either as they are or after some form of regeneration [17]. The types of heterogeneous catalyst can be further categorised by their support, with examples including polymers, dendrimers, mesoporous materials, zeolites, metal oxides, and carbon.

Reactions using palladium supported on mesoporous materials have been reported by numerous authors. Among them, Zhao et al. [18] studied the coupling of non-activated aryl iodides with different alkenes using Pd-MCM-41-NH_2_. Other examples include Pd-FSM 16 (functionalised with pyridine-carboimine or quinoline-carboimine) [19], oxime carbapalladacycle anchored on several mesoporous materials [20], and a palladium bipiyridyl complex anchored on nanosized MCM-41 [21]. Furthermore, a myriad of zeolites have been applied in Heck reactions, with examples including: modernite, HY or beta [22–23], montmorillonite [24], layered double hydroxides (LDH) [25]. Heterogeneous catalysts also lend themselves particularly well to continuous processing. One such example is reported by Karbass et al. [26], who carried out continuous flow reaction using Pd(0) derived from [Pd_3_(OAc)_6_] supported on polymeric monoliths containing methylimidazole, an ionic-liquid (IL) moiety in near-critical ethanol. It is thought that the resins act as catalysts to release active palladium-species into solution. It also appeared that the IL-like unit was capable of capturing and stabilising the catalytically active soluble palladium species that are generated during the reaction. The reaction system eliminated the requirement of an inert atmosphere. Reactions between iodobenzene and methyl acrylate achieved 85% yield and 100% selectivity to *trans*-methyl cinnamate at 200 °C and 80 bar. The reaction system was then extended to acrylonitrile as the olefinic substituent, and achieved a yield of 66% and 70% *trans*-selectivity under similar conditions [26]. Other examples of carbon–carbon cross-coupling reactions utilising continuous flow systems were also reported. However, conventional organic solvents were required either during the reaction or for the extraction progress [3–4,26–31].

The majority of the examples of catalysts discussed employ phosphines as electron-donating ligands but this is undesirable as they are toxic, air-sensitive, and moisture-sensitive [32]. An excess amount of phosphine and palladium are normally required under the Heck reaction conditions as they are susceptible to decomposition. Unfortunately, an excess of phosphine reduces the reaction rate, while higher quantities of palladium result in potentially significant increased production costs [33]. Palladium on a magnesium–lanthanum mixed oxide support (Pd/MgLaO) was reported to be an efficient, thermally stable and highly active heterogeneous catalyst in the Heck reactions of styrene and halides, including iodides, chlorine substituents, and both activated and non-activated bromine [32]. It has been rationalised that the MgLaO support itself appears to act as a base, which in turn acted as an electron-donating group relative to Pd, thereby eliminating the requirement of the phosphine ligand. The catalyst can also aid the reaction without requiring an inert atmosphere.

In this paper we report the Heck reaction between 4-iodoanisole and either styrene or methyl acrylate in a continuous flow reactor. The solvent system used was a mixture of scCO_2_ and THF/methanol which contained the alkene and the organic base, DIPEA. In order to reduce the complexity of the system, palladium metal supported on silica was used as catalyst and no phosphines were added. While this was likely to reduce the activity of the system over homogeneously catalysed reactions using complexes with elaborate ligand design, it would also make purification simpler.

## Results and Discussion

**Scheme 1:**
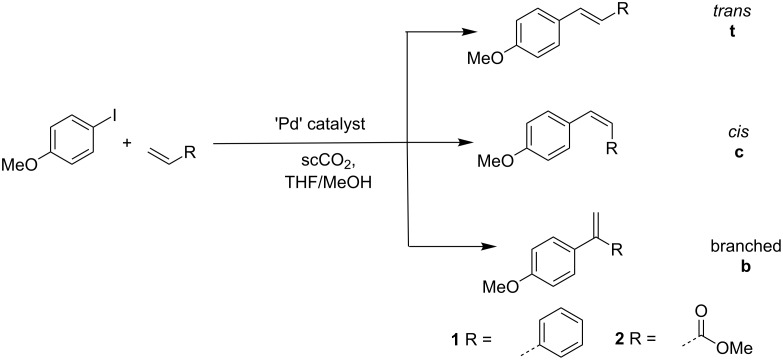
General Heck reaction showing the possible isomers that can be produced.

Two reactions were studied using continuous flow chemistry as shown in Scheme 1. In both cases 4-iodoanisole was used as the aryl halide. The alkene used was either styrene to yield the stilbenes **1** or methyl acrylate to yield the cinnamate ester **2**. For each reaction there are three possible regioisomers. The thermodynamically favoured *trans*- (**t**) isomer is most common in standard organic solvents although under certain conditions the *cis*- (**c**) isomer can be formed under kinetic control. The third possibility is the *geminal* or branched (**b**) isomer which is usually formed where a cationic intermediate forms that facilitates migration of the σ-Pd–C bond from the terminal alkenic 1-position to the 2-position. In the majority of publications, the formation of the branched isomer is either not observed or not reported. Certainly for activated alkenes the branched isomer rarely forms [12], however we have reported previously that it can be observed in both homogeneous and heterogeneously catalysed reactions and also under conditions of continuous flow [5].

### Synthesis stilbenes under stirred tank and continuous flow conditions

**Figure 1:**
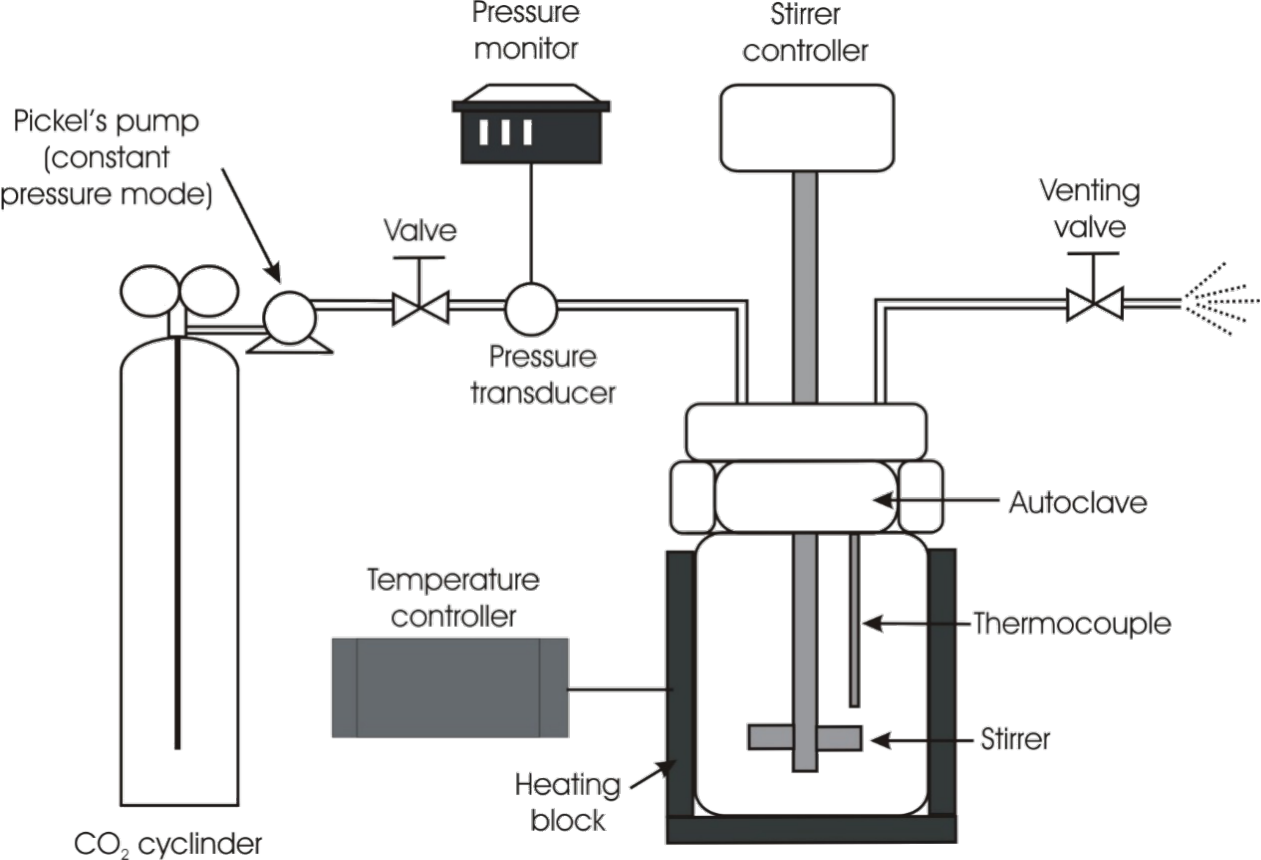
Schematic diagram of the stirred autoclave reactor. Pickel’s pump NWA PM101 was used to achieve supercritical pressure.

The synthesis of 4-methoxystilbene (**1**) was carried out under continuous flow conditions and also in a stirred autoclave reactor to compare the processes. In the autoclave reaction (Figure 1) a single composition was investigated corresponding to the concentrations in the continuous flow reaction set for a scCO_2_/organic solution ratio of 5:1. The pressure of the autoclave was set as close to 200 bar as possible, however because no back pressure regulator was present this was achieved by setting the applied pressure at 167 bar then increasing the temperature to 145 °C to attain reaction temperature and a pressure close to 200 bar. Because the autoclave is a sealed system it was difficult to take samples on a regular basis. Therefore, the reactor was run for 24 hours and the sample then analysed after de-pressurising the system. A total conversion of 94% was obtained (GC analysis relative to the limiting reagent 4-iodoanisole), with 77% conversion to the **t** isomer and 17% to the **b** isomer. No **c** isomer was detected. This gave a **t**/**g** ratio of 4.5 to 1. The remaining analysis corresponded to unreacted 4-iodoanisole.

### Continuous flow reactions

**Figure 2:**
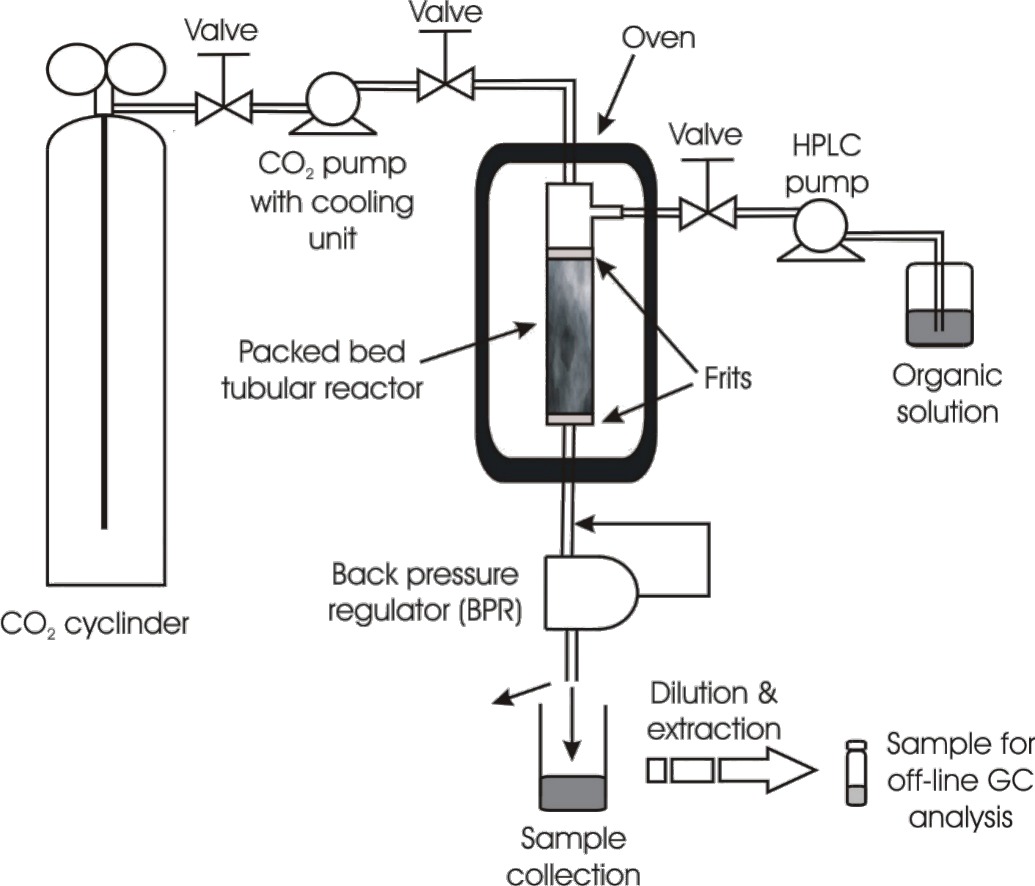
Schematic diagram of the continuous flow system. The reactor shown is the 3.9 mm i.d. PFR. For the 1 mm i.d. reactor this was a coil of the 300 cm or 100 cm tube according to the specific reaction.

All reactions were performed in 300 or 100 cm stainless steel tubular plug flow reactors (PFRs) of 1 mm internal diameter. A larger diameter (3.9 mm) PFR was also used as a comparison in one example (Figure 2). Initial studies used flow rates commensurate with the concentration used in the autoclave reactor.

### Residence time in the continuous flow reactors

The volume of the unpacked 1 mm diameter reactors in the styrene and methyl acrylate reactions were 2.36 and 0.79 mL respectively. The reactors containing catalyst with packing were found to have a porosity of 0.80, so neglecting packing swelling, the total volumes available for flow for the 300 cm and 100 cm length reactors were 1.89 and 0.63 mL respectively. Unless otherwise stated, the scCO_2_ flow rate was 0.1 mL min^−1^ while the organic flow rate was precisely varied over the range shown in Table 1 to give the total flow rates shown. The range of possible residence times on each reactor are also given in Table 1, based on the total flow rates and effective reactor volumes.

**Table 1:** Correlation of reaction flow rates to reactor residence times.

Flow rates, mL/min			Residence times, min	
scCO_2_	Organic	Total	300 cm reactor	100 cm reactor

0.1	0.033	0.133	14.2	4.7
0.1	0.025	0.125	15.1	5.0
0.1	0.02	0.12	15.7	5.2
0.1	0.017	0.117	16.1	5.4
0.1	0.014	0.114	16.5	5.5
0.1	0.013	0.113	16.7	5.6
0.1	0.011	0.111	17.0	5.7

Because of the piping pre- and post-reactor there was a volume through which flow was possible but without reaction. Therefore, after the commencement of organic solution flow, samples were not collected over the initial 2 hour period to allow transit of the fluids through the reactor system. The reactor was operated over an eight hour period taking the first sample at 3 hours unless otherwise shown and then at regular intervals.

**Figure 3:**
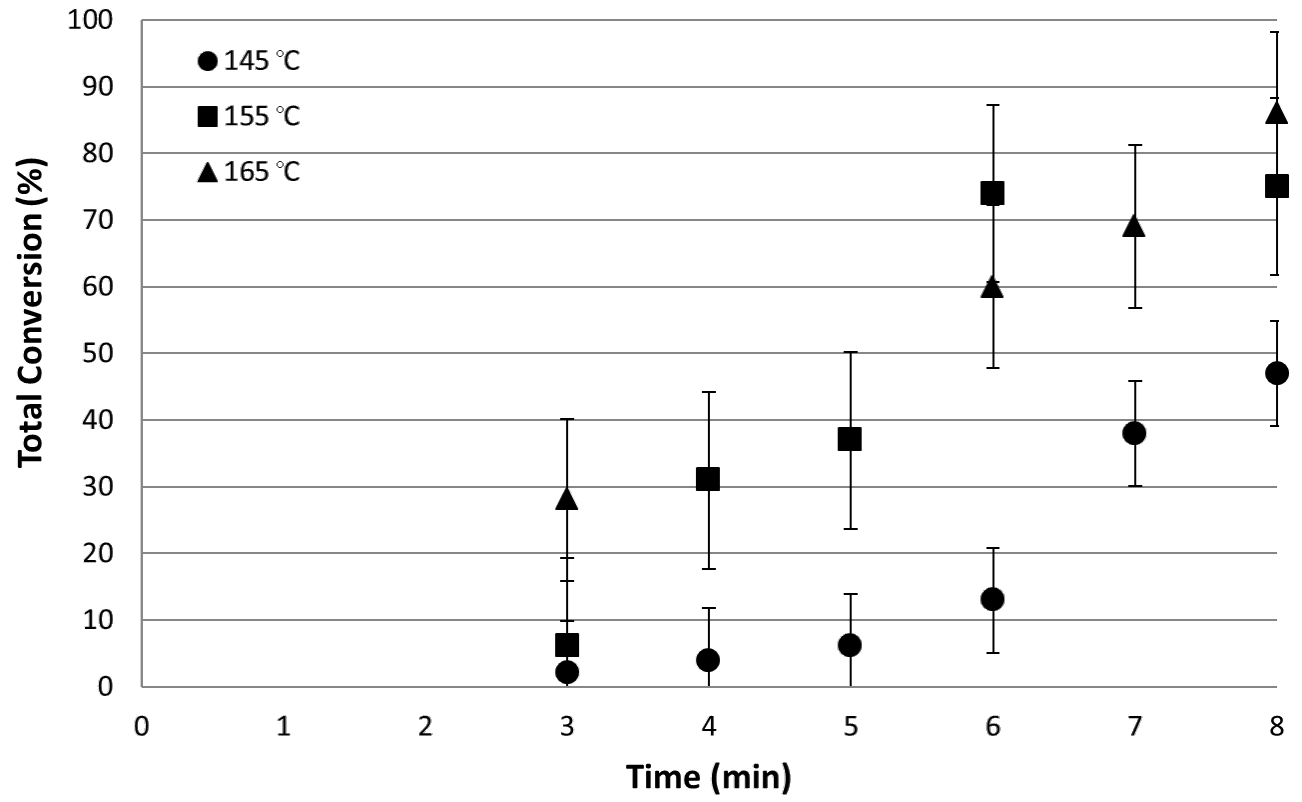
Total conversion of 4-iodoanisole as a function of reactor run time for three reaction temperatures.

In the initial flow studies, the scCO_2_ flow rate was set at 0.1 mL min^−1^ and the organic solution flow rate at 0.02 mL min^−1^. The temperature was controlled to ±0.1 °C using a Memmert oven. Three reaction temperatures were chosen: 145, 155 and 165 °C. The first samples were analysed 3 hours after the organic flow was started to allow complete flushing of the complete reactor system with reagents. Samples were then taken at regular intervals over an eight hour period. Figure 3 shows the total conversions obtained for combined **t** and **b** isomers. No **c** isomer was observed in any of the reactions carried out.

The first feature of note is that the initial conversion at all temperatures is low. The system takes time to reach equilibrium as the catalyst becomes conditioned so therefore requires a start-up procedure before reaching full conversion capacity.

At 145 °C, conversion of 47% is achieved with a **t**/**b** ratio of 6:1 although the system is far from running at equilibrium conversion. Curve fitting shows that after 24 hours approximately 60% conversion would be achieved. Therefore on comparable timescale the continuous flow reactor gives a lower conversion but with a slightly improved selectivity for the conversion to the **t** isomer. Increasing the temperature to 155 °C resulted in 75% conversion after 8 hours with a **t**/**b** ratio of 4:1. At 165 °C the conversion was 86% after 8 hours although the **t**/**b** ratio has decreased to 3:1.

**Figure 4:**
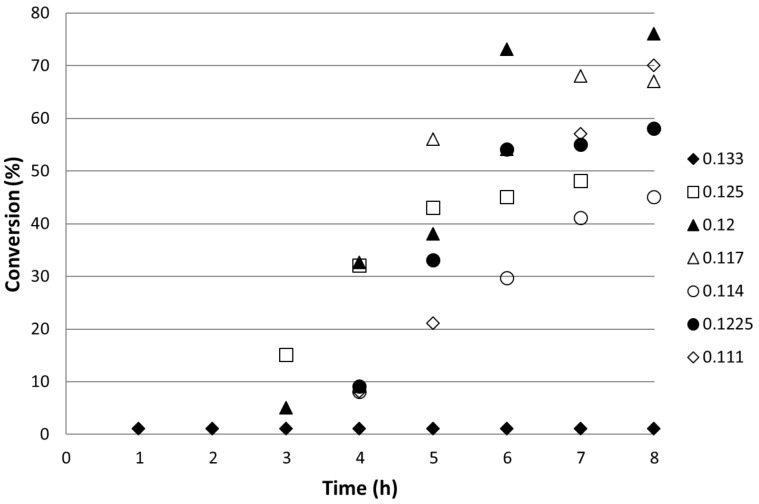
Total conversion of 4-iodoanisole at 155 °C and 200 bar as a function of reactor run time for different combined flow rates and therefore residence times. The values of the total flow rates given in the legend are mL min^−1^.

The effect of overall flowrate through the reactor was determined by changing the organic flow rate while keeping that of the scCO_2_ constant at 0.1 mL min^−1^. Therefore, the concentration of the organic reagents increased with increased flow. The temperature was maintained at 155 °C and the pressure at 200 bar throughout. Figure 4 shows the reaction profile for the rates studied. Optimum conversion of 76% was observed at 0.12 mL min^−1^. At faster flows the conversion decreased with the lowest conversion (1%) was observed for the fastest flow rate of 0.133 mL min^−1^. At slower flow rates the conversion decreased away from the optimum value showing the process to be a compromise between flow rate and reaction rate.

**Figure 5:**
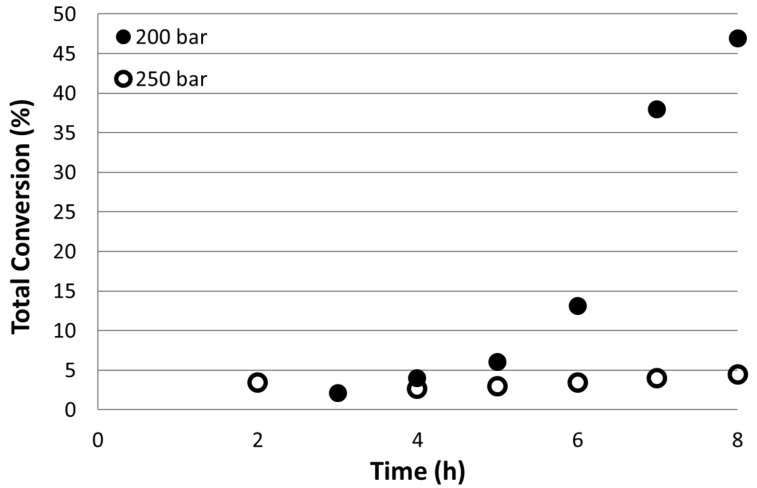
Total conversion of 4-iodoanisole as a function of reactor run time at 145 °C and at 200 (●) and 250 (ο) bar CO_2_ pressure.

Increasing the pressure from 200 to 250 bar had a dramatic effect on conversion. The continuous flow reaction was repeated at 145 °C and flow rate of 0.12 mL min^−1^ but at 250 bar scCO_2_ pressure. The results are shown in Figure 5. While the conversion increased to 48% at 200 bar with continued use until the reaction was stopped, there was less than 5% conversion in all cases when the pressure was increased to 250 bar. At this time we cannot account for this almost complete inhibition of the reaction at increased pressure so we are carrying out further studies to elucidate the precise mechanism. We are also investigating the effect of pressure over an extended range to consider solutions at sub- and near-critical pressures to determine the optimum conditions for conversion and selectivity in continuous flow systems. Furthermore, it was noted that while there was a dramatic change in conversion, the **t**/**b** ratio remained unchanged at 6:1.

### Synthesis of methyl 4-methoxycinnamate under continuous flow conditions

An activated alkene, methyl acrylate was reacted with 4-iodoanisole under catalytic PFR conditions and the conversion and product distribution monitored. A column containing the 2% Pd/SiO_2_ catalyst with Chromosorb packing (1:3) was constructed with dimensions 100 cm × 1 mm by cutting fresh sections of the 300 cm tubes used for the reactions previously described using styrene. Each reactor therefore contained 15.7 μmol Pd dispersed throughout the volume. The pressure was maintained at 200 bar and 155 °C throughout and the total flow rate was set to be 0.12 mL min^−1^ using a scCO_2_ flow rate of 0.1 mL min^−1^. The residence time on the reactor was calculated as 5.2 minutes. Including flow in the upstream and downstream pipework then the total time on the system was 84 minutes so the first sample was taken after 90 minutes.

**Figure 6:**
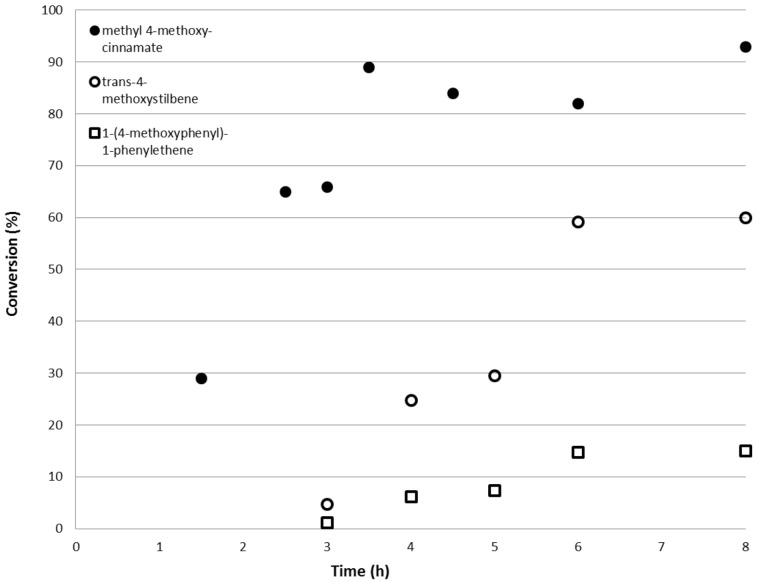
Conversion of 4-iodoanisole to methyl 4-methoxycinnamate (●) at 155 °C and 200 bar as a function of reactor run time. The open symbols are the conversions to the different isomeric products for the reaction of 4-iodoanisole with styrene for comparison.

Figure 6 shows that reaction between 4-iodoanisole and methyl acrylate proceeded more rapidly than the reaction with styrene under the same operational conditions. The conversion reached equilibrium after only 3.5 hours with a conversion of 4-iodoanisole of 90 ± 3%. Comparisons between methyl acrylate and styrene also shows that the latter requires a reaction run time of about 6 hours to achieve comparable yields and conversion. The increased reactivity of methyl acrylate over styrene can be explained in terms of the increased resonance stabilisation of the intermediate through electron delocalisation over the ester carbonyl motif.

Another aspect of interest is the product selectivity in the reactions involving 4-iodoanisole and methyl acrylate. The terminal product, methyl *trans*-methoxycinnamate (**2t**) was obtained exclusively, whereas in the system using styrene, a mixture of the trans-linear (**1t**) and terminal-branched (**1b**) isomers were observed with the composition dependent on the system temperature. This may be explained due to a steric effect [34]. In the case of methyl acrylate, proper alignment of the double bond for migratory insertion is hindered by the presence of the methyl group, thereby favouring the formation of the terminal arylation product. The presence of the methyl group on methyl acrylate might have a more profound impact on steric limitation than the phenyl group of styrene. The results shown here are consistent to published literature utilising 4-iodoanisole and methyl acrylate in the presence of ruthenium- and platinum-complex catalysts, in which complete regioselectivity towards the *trans*-isomer was achieved [19].

Having obtained encouraging conversions in the 1 mm diameter reactor, it was decided to scale the reaction out to a 3.9 mm internal diameter PFR. The 9 cm long reactor was packed with the catalyst and packing material and the reaction carried out under identical reaction conditions to those used in the 1 mm reactor. Accounting for the 0.80 porosity of the reactor filled with the packed catalyst, the total available volume for fluid flow in the 3.9 mm reactor was 0.88 mL with a residence time at a combined flow rate of 0.12 mL min^−1^ of 7.3 minutes.

**Figure 7:**
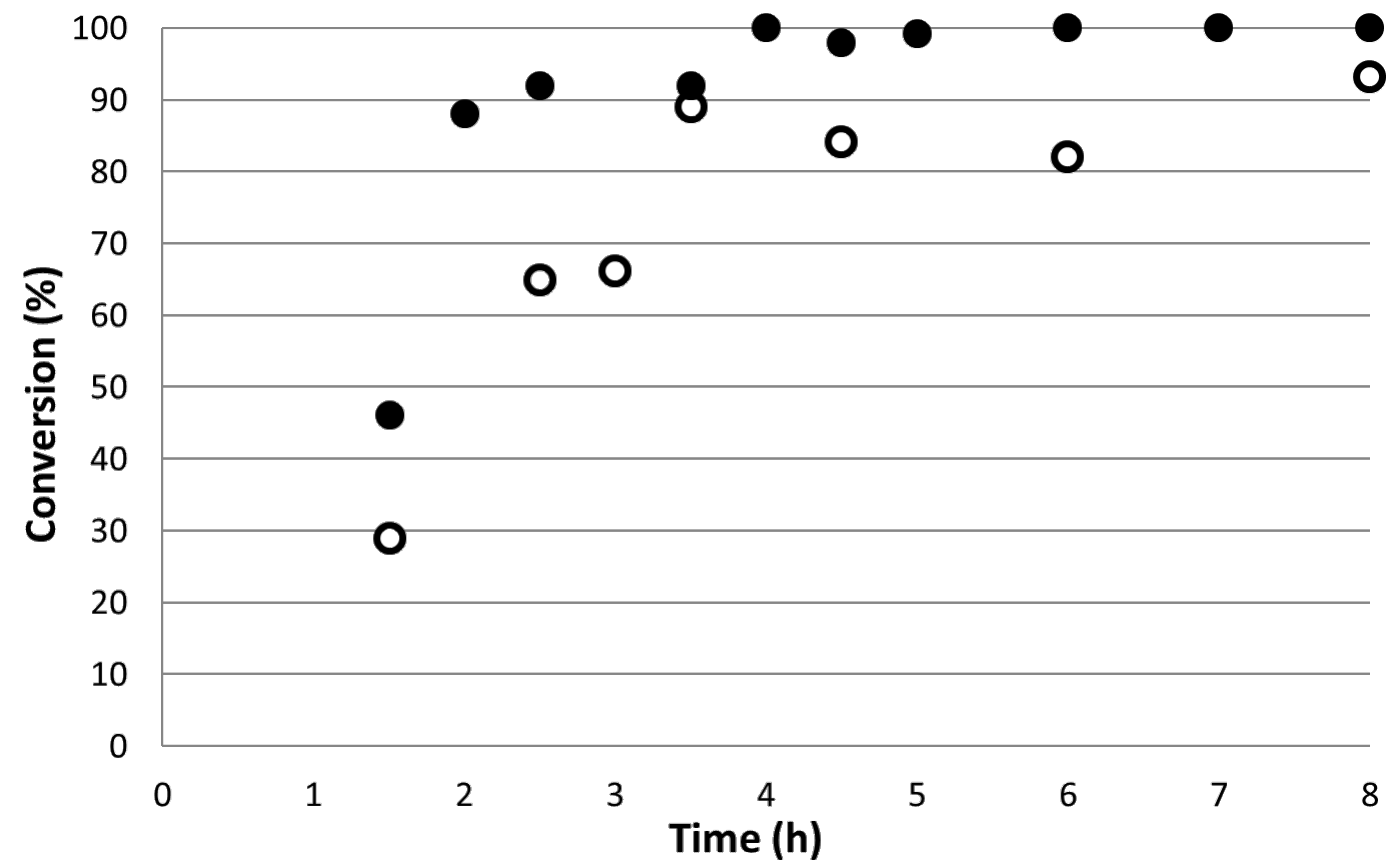
Comparison of conversion as a function of reactor run time for the reaction of methyl acrylate in the 1 mm × 300 cm (ο) and 3.9 mm × 9 cm (●) plug flow reactors over the Pd-SiO_2_ catalyst.

**Figure 8:**
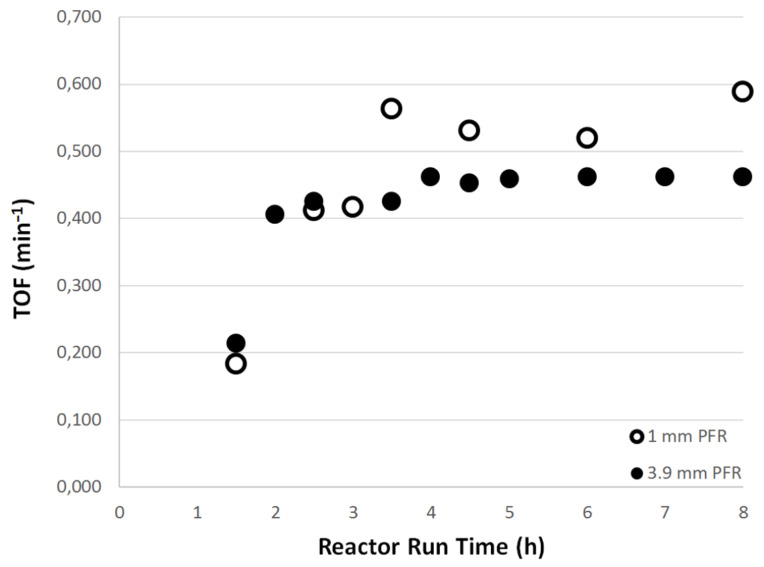
TOF values for the 1 mm (ο) and 3.9 mm (●) PFRs at a total flow rate of 0.12 mL min^−1^, 155 °C and 200 bar for the reaction of 4-iodoanisole with methyl acrylate.

Figure 7 shows that the yields obtained in the 1 mm PFR are slightly lower than those achieved in the 3.9 mm reactor, however the mass of catalyst was also lower. Furthermore, the 3.9 mm PFR gave complete conversion of the starting material after only 4 hours flow and this conversion was maintained while flow continued. In order to study the effects of the catalyst amount, the turnover frequency (TOF) was also calculated and this is shown in Figure 8. It is clear that within experimental error that the TOFs in the two reactor configurations are essentially identical which is encouraging in terms of the ability to both scale up and scale out the reactions. The TOF is defined as the number of moles of substrate converted per mol of catalyst per unit time. For a continuous flow system this can be defined in terms of the molar or volumetric flow rates (*u*_M_ or *u*_V_), the conversion as a mol fraction (*x*) and the number of mol of substrate at the start of the reaction (*n*_S_) and the number of mol of catalyst (*n*_CAT_) as shown in Equation 1.

[1]
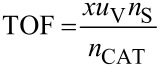


**Figure 9:**
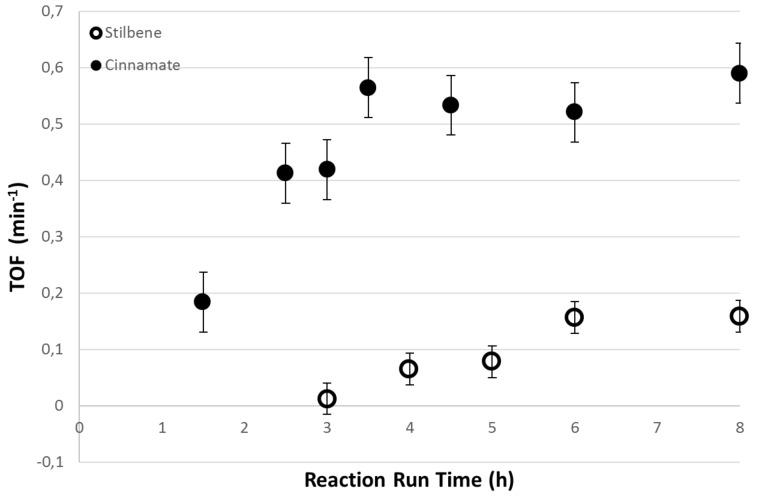
Turnover frequencies of the Heck reactions at 155 °C using styrene (ο) as combined isomeric products, and methyl acrylate (●) as a function of reactor run time for the 1 mm internal diameter plug flow reactors.

The TOFs were also calculated for the reactions of styrene and methyl acrylate with 4-iodoanisole in the 1 mm PFRs in order to normalise with respect to catalyst concentration as the lengths of the reactor and therefore residence times for the same flow rate differed three-fold. The results are shown in Figure 9 and emphasise the more reactive nature of the methyl acrylate as a substrate. Indeed the methyl acrylate is almost four times more reactive than styrene under identical conditions of temperature and pressure.

## Conclusion

Supercritical carbon dioxide (scCO_2_) can be effectively used as a reaction solvent for the palladium metal-catalysed Heck reaction between an aryl halide and an alkene under continuous flow conditions in a plug flow reactor (PFR). The efficiency of the reaction depends on the nature of the alkene used, the temperature and pressure of the reaction and the flow rate of the reagents over the fixed bed catalyst. It was found that increasing the temperature of the reaction between 4-iodoanisole and styrene resulted in an increased conversion but that the distribution of isomers was affected with more of the branched, germinal addition isomer being produced at higher temperatures. In the case of reaction with methyl acrylate as the alkene, only the single *trans-*substituted isomer was produced. Increasing the pressure from 200 to 250 bar in the PFR had the dramatic effect of almost completely inhibiting the reaction. The turnover frequency is, as expected, considerably higher when methyl acrylate is used instead of styrene. However, there is very little change in the TOF for methyl acrylate when the internal diameter of the PFR was increased from 1.0 to 3.9 mm. This is encouraging for the design of continuous flow reactor systems as the TOF is essentially insensitive to diameter scaling over the length scales studied and hence there are good prospects for scaling the flow to handle bigger substrate inventories.

## Experimental

### Reagents and instruments

All reagents were purchased from Sigma-Aldrich unless otherwise stated and were used as received without further purification. The supported catalyst, 2% palladium on silica was purchased from Johnson Matthey.

Gas chromatography–mass spectra GC–MS analyses (EI and CI modes) were performed using a PerkinElmer GC–MS with a 30 m × 0.25 mm × 0.25 μm Phenomenex-ZB5 column by Jane Stanbra and Simon Thorpe in the Department of Chemistry, The University of Sheffield. The oven temperature programme was 60–260 °C at 10 °C/min with a final temperature isothermal hold for 10 minutes, corresponding to a total run time of 30 minutes. The MS mass limit was set at between 50 and 450 Da. GC analyses were carried out using a Varian 3900 GC fitted with an autosampler (AS 8400) and a flame ionisation detector (FID) on a 15 m × 0.25 mm × 0.25 μm CP-Sil 5CB Chrompak Capillary Column. The oven temperature programme was set to ramp from an initial temperature of 50 °C (held for 1 minute) to 250 °C (held for 0.5 minutes) at a rate of 20 °C/minute, totalling to a run time of 11.5 minutes. The temperatures of the injector and the FID were set at 200 and 250 °C respectively.

Inductively coupled plasma-atomic emission (ICP-AE) analyses were performed on a Spectro Ciros CDD instrument (SpectroAnalytical, UK) by Alan Cox and Neil Bramall, Department of Chemistry, The University of Sheffield. The catalyst particles were rinsed with organic solvents (diethyl ether, THF and methanol, 2 × 5 mL each), followed by distilled water (2 × 5 mL) and dried under vacuum prior to analysis. During the sample preparation step, the catalyst sample was quantitatively transferred to a clean (acid-washed) tube and 6 mL of concentrated hydrochloric acid and 2 mL of concentrated nitric acid were added. The sample was placed in a dri-block which was thermostatically controlled with the temperature gradually raised to 150 °C. The sample was then maintained at this temperature for 1 hour. After this digestion period, the sample was left to cool and quantitatively transferred to a 50 mL tube and topped up to 50 mL with 1% nitric acid. The element of interest (palladium) was measured against a calibrated sample prepared from known standards. The sample was introduced to the instrument using a flow regulated pump. On reaching the nebuliser, the liquid sample met argon gas, generating a spray of droplets. The heavy droplets were captured by the spray chamber walls and were pumped to a waste container. The fine droplets (ca. 2% of the sample) passed through the spray chamber and into the argon plasma (ca. 7000 °C) via the torch. The resulting atoms and ions were excited and emitted light of characteristic wavelengths (Pd measured at 340.458 nm). The light was detected by charged coupled devices (CCD chips) mounted on the spectrometer. The concentration of Pd was then calculated using the instrument software. ICP–AE analysis gave the concentration of palladium metal on the silica gel support as 0.02 g or 0.189 mmol Pd/g. This was later diluted with Chromopac for use in the flow reactors at a ratio of 1:3 catalyst/packing.

### Stirred autoclave reactions

The autoclave reaction set-up is shown schematically in Figure 1. A standard reaction solution made from 4-iodoanisole (1 equivalent, 3.5105 g, 15 mmol), styrene (2 equivalents, 3.1245 g, 30 mmol), *N*,*N*-diisopropylethylamine (DIPEA, 2 equivalents, 5.23 mL, 30 mmol) and dissolved in THF (15 mL) and methanol (15 mL each) was prepared and stirred thoroughly until completely mixed. The ratio of organic to scCO_2_ used in the autoclave corresponded to the 5:1 volumetric ratio used in the continuous flow reactions. The tetrahydrofuran and methanol were used to dissolve the organo iodide and act as a miscible, polar modifier to enhance the solvating properties of the scCO_2_. The 60 mL autoclave was charged with 2.4 mL of the reaction solution, corresponding to 4% of the autoclave volume. 2% Palladium on a silica support catalyst (20.4 mg, 2.463 mmol) was then added and the autoclave sealed and inserted into a pre-heated aluminium heating block (external temperature 145 °C, internal temperature 120 °C). After a period of 30 minutes the remaining reactor volume (57.6 mL) was pressurised with scCO_2_ at 167 bar using a Pickel’s pump NWA PM-101 operating in constant pressure mode. The reaction was stirred for 24 hours at a rate of 145 rpm. To work-up the reaction mixture, the autoclave was allowed to cool to room temperature and then slowly depressurised by venting the CO_2_. The reaction mixture remaining in the autoclave was then dissolved in additional tetrahydrofuran, before extracting into diethyl ether and washing with water. The resulting organic mixture (top layer) was then withdrawn and dried over magnesium sulfate then analysed by gas chromatography using a Varian 3900 GC with reference to standard solutions of *trans*-4-methoxystilbene.

### Continuous flow reactions

Particles of the catalyst, 2% Pd on silica, (250 mg) thoroughly mixed with the packing material (Chromosorb 750; 100–120 mesh; 750 mg) were packed into standard HPLC columns (length 300 cm, internal diameter 1 mm). The columns were laid out straight and a small pressure (1 bar) was applied to fill the material into the column. No vacuum was used. The catalysts were dispersed in a uniform manner through the whole length of the column, and finally the ends plugged with 25 μm stainless steel frits. For the reaction with methyl acrylate, the column was cut into 100 cm sections. The unpacked volume of the 300 cm long reactor was 2.36 mL and the 100 cm long reactor 0.79 mL. In all cases the columns were then coiled a small volume within the cavity of a Memmert oven. The setup for the continuous flows is shown schematically in Figure 2.

The reactor was connected to the fluid delivery system using standard Swagelok fittings, and housed in a Memmert oven with custom fitted inlet and outlet ports. Supercritical carbon dioxide was generated by passing the feed from a CO_2_ cylinder (BOC, liquefied at 15 °C, 50 bar pressure) through a Jasco PU-1580-CO2 supercritical pump. The system was first saturated with CO_2_ at high flowrates (1 to 2 mL) until the desired pressure was reached, followed by gradual reduction of the CO_2_ flowrate to the target value. The system was heated up when the reactor was saturated with CO_2_ at 200 bar. When the desired temperature was reached, the system was left to equilibrate for a further period of 15 minutes. Pumping the organic reaction mixture (4-iodoanisole, 15 mmol; styrene, 30 mmol; *N,N*-diisopropylethylamine, 30 mmol; tetrahydrofuran, 15 mL; methanol, 15 mL) was achieved using a Jasco PU-1580 HPLC pump set at constant flow mode. The organic reaction mixture was pumped at a variety of flowrates while maintaining constant scCO_2_ flowrate of 0.1 mL/min for all runs. The entire system pressure was controlled by a Jasco PU-1580-81 back pressure regulator (BPR). Samples were collected at regular intervals using vials placed underneath the BPR outlet port. The CO_2_ evaporated immediately on de-pressurisation leaving a solid residue containing the mixture of products. The samples were then analysed by gas chromatography (GC) and GC-mass spectrometry (GC–MS) against known calibration standards of the starting material and products. At the end of reaction, the system tubing and reactor were flushed with CO_2_ and the organic solvent mixture (THF and methanol) for a further period of 6 hours to remove any products, byproducts and unreacted organic reagent.

A larger continuous flow reactor with 3.9 mm internal diameter of length 9 cm was also used as a comparison to assess the effects of scaling up the flow reactor. This was constructed from stainless steel tubing with 25 μm steel frits at each end, connected to the solvent delivery system using standard ¼ inch Swagelok fittings. The reactor body was filled with the 1:3 (w/w) mixture of the 2% palladium on silica catalyst and Chromosorb packing material. The unpacked volume of the reactor was 1.075 mL.

### Sample analysis

To work up the reaction, the raw sample was diluted with diethyl ether (2 × 3 mL), then washed with an equivalent volume of distilled water to dissolve the halide salt formed. Diethyl ether (2 mL) was added to a GC vial and 5 drops of solution from the organic top layer of the extraction mixture was withdrawn using a Pasteur pipette and added into the GC vial. The solution was thoroughly mixed and analysed by GC and/or GC–MS.
